# Cherries with Different Geographical Origins Regulate Neuroprotection in a Photoperiod-Dependent Manner in F344 Rats

**DOI:** 10.3390/antiox13010072

**Published:** 2024-01-03

**Authors:** Francesca Manocchio, Francisca Isabel Bravo, Gisela Helfer, Begoña Muguerza

**Affiliations:** 1 Nutrigenomics Research Group, Department of Biochemistry and Biotechnology, Universitat Rovira i Virgili, C Marcel·lí Domingo s/n, 43007 Tarragona, Spain; francesca.manocchio@upf.edu (F.M.); begona.muguerza@urv.cat (B.M.); 2 Nutrigenomics Research Group, Institut d’Investigació Sanitària Pere Virgili, C/Marcel·lí Domingo s/n, 43007 Tarragona, Spain; 3 Center of Environmental, Food and Toxicological Technology (TecnATox), Universitat Rovira i Virgili, 43007 Tarragona, Spain; 4 School of Chemistry and Biosciences, University of Bradford, Richmond Road, Bradford BD7 1DP, UK

**Keywords:** phenolic compounds, antioxidant-rich fruit, hippocampus, neurotroph, BDNF, seasonal, photoperiod

## Abstract

The photoperiod is the main environmental cue that drives seasonal adaptive responses in reproduction, behavior, and metabolism in seasonal animals. Increasing evidence suggests that (poly)phenols contained in fruits can also modulate seasonal rhythms. (Poly)phenol-rich diets are associated with an improvement in cognitive function and neuroprotection due to their anti-inflammatory and antioxidative properties. However, it is unknown whether cherries affect neuroprotection in a photoperiod-dependent manner. To test this, F344 rats were exposed to L6 (6 h light/day), L12 (12 h light/day) and L18 (18 h light/day) photoperiods and fed a standard chow diet supplemented with either a control, lyophilized cherry 1 or cherry 2 with distinctive phenolic hallmarks. Physiological parameters (body weight, eating pattern index (EPI), testosterone, T4/T3) and hypothalamic key genes (*Dio2, Dio3, Raldh1* and *Ghrh*) were strongly regulated by the photoperiod and/or fruit consumption. Importantly, we show for the first time that neurotrophs (*Bdnf, Sod1* and *Gpx1*) in the hippocampus are also regulated by the photoperiod. Furthermore, the consumption of cherry 2, which was richer in total flavonols, but not cherry 1, which was richer in total anthocyanins and flavanols, enhanced neuroprotection in the hippocampus. Our results show that the seasonal consumption of cherry with a specific phenolic composition plays an important role in the hippocampal activation of neuroprotection in a photoperiod-dependent manner.

## 1. Introduction

Many organisms adapt to seasonal fluctuations in the climate with profound changes in their physiology, behavior and reproduction [1]. These seasonal changes are driven by changes in day length (photoperiod) and mediated via neuroendocrine processes in the hypothalamus. Briefly, photoperiod information is biochemically translated through the release of melatonin from the pineal gland during dark hours. Melatonin then acts on the pars tuberalis of the pituitary gland to regulate thyroid-stimulating hormone release, which, in turn, regulates the thyroid hormone availability of the deiodinase enzymes DIO2 and DIO3 in the hypothalamic tanycytes lining the third ventricle of the hypothalamus. Indeed, the hypothalamic tanycytes express thyroid-stimulating hormone receptors. Thus, they sense thyroid-stimulating hormone subunit β signals from the pituitary, expression of which is upregulated by the photoperiod and results in long-photoperiod-induced *Dio2* mRNA expression (12, 14 or 16 h of light) and short-photoperiod-induced *Dio3* mRNA expression (6 and 8 h of light). DIO2 converts the inactive form of the thyroid hormone, thyroxine (T4), into the active form, triiodothyronine (T3), increasing the hypothalamic concentration of T3 [1]. Thus, the hypothalamic–pituitary–thyroid and hypothalamic–pituitary–gonadal axes are sensitive to changes in photoperiod [2]. In addition, hypothalamic tanycytes express a variety of nutrient sensors [3]. Nutrients (glucose, amino acids and lipids), as well as hormones (leptin, ghrelin and insulin) produced in response to nutrient availability, can modulate gene expression in tanycytes, which ultimately regulates energy metabolism [4]. Moreover, the hypothalamus is sensitive to phenolic compounds, which are among the main bioactive components of fruits and vegetables [5]. In support, the administration of grapes and cherries, which are rich in phenolic compounds, to photoperiod-sensitive Fischer 344 (F344) rats increases hypothalamic leptin sensitivity when they are consumed during a short photoperiod [5,6]. Phenolic compounds can exert a neuroprotective effect. A recent study showed that the consumption of tart cherries, a fruit rich in phenolic compounds, significantly affects the plasma metabolome, associated with an improvement in cognitive function in humans consuming this fruit [7]. For example, tart cherry exerts anti-inflammatory properties, suppresses neuronal apoptosis and stimulates pro-survival signaling cascades in vitro [8] and in aged rats [7,8]. Furthermore, aged rats consuming blueberries, a fruit also rich in phenolic compounds, show an improvement in working memory and an upregulation of hippocampal brain-derived neurotrophic factor (*Bdnf*) gene expression compared with control rats [9]. The BDNF protein plays a key role in neuronal plasticity, and, together with nuclear factor erythroid-derived 2-like (NRF2), promotes the gene expression of antioxidant enzymes in the hippocampus [10].

Recently, our group showed that Royal Dawn sweet cherries increased serum and hepatic antioxidant markers in F344 rats depending on the cherry composition profile and the photoperiod of consumption [11]. Moreover, consumption of the same sweet cherries modulated the activity of transaminase enzymes and reduced lipid peroxidation markers in Fisher 344 rats, also depending on their phenolic profile and the photoperiod in which they were consumed [11]. Cherries are considered a good source of dietary phenolic compounds [12,13]. The predominant phenolic compounds are cyanidin derivatives (anthocyanins), with cyanidin-3-*O*-rutinoside being the major compound [14,15]. Moreover, cherries also contain high levels of hydroxycinnamic acids, caffeoylquinic acid derivatives and rutin, although their profiles can differ depending on the method used to extract the cherry’s phenolic fraction [15]. Interestingly, as (poly)phenols are plant stress metabolites, the cherry’s phenolic profile is distinctive and depends on the location, time of harvest and storage conditions, among other factors [16,17]. Although cherries are seasonal fruits (available from May to August), out of season they are harvested and imported from different geographical locations to allow for continuous sales and consumption [18]. However, it is yet unknown whether the consumption of cherries with different phenolic compositions exerts a neuroprotective effect in a photoperiod-dependent manner, reducing oxidative stress. The aim of this study was to investigate if the consumption of sweet cherries plays a role in neuroprotection in a photoperiod- and composition-profile-dependent manner.

## 2. Materials and Methods

### 2.1. Fruit Preparation and Composition

Royal Dawn sweet cherries (*Prunus avium* L.) were harvested in two different geographical locations in order to obtain cherries with different compositions. Cherries from the Tarragona region in Spain (Ch1) were kindly donated by a local farmer. Cherries from Chachapoal in Chile (Ch2) were purchased in a local market. Sweet cherry pulps were frozen in liquid nitrogen, ground and lyophilized. After lyophilization, the obtained cherry powders were kept at −20 °C until administration to animals. Basic composition was analyzed according to the AOAC method [19], and the phenolic content of these two cherries has been published in Cruz-Carrión et al. 2020 [11]. Phenolic compounds in the cherries were extracted for analysis using 65% methanol (1% formic acid) at a ratio of 80 mL/g of cherry, 72 °C, and 100 min of agitation at 500 rpm [11,15]. Phenolic composition was analyzed using spectrophotometric methods, total polyphenols by the Folin–Ciocalteu method, total anthocyanins by the pH differential method, total flavanols by the DMACA method, and total flavonols by mixing the sample with 0.1% HCl and measuring the absorbance at 360 nm after 15 min of reaction [15,20,21]. Ch1 showed higher contents of total ash, protein, lipids, fiber, polyphenols, anthocyanins and flavanols than Ch2; however, Ch2 showed higher levels of total carbohydrates, sugars and total flavonols than Ch1 [11]. Additional HPLC-ESI-MS/MS studies showed that anthocyanins were the major phenolic family in both cherries (75 and 52% of the total quantified phenolic compounds for Ch1 and Ch2, respectively), followed by flavonols, flavanols and phenolic acids. The major compounds were cyanidin-3-*O*-rutinoside (Ch1 > Ch2, 3.42 times), quercetin-3-*O*-rutinoside (Ch2 > Ch1, 1.18 times), catechin (Ch1 > Ch2, 3.20 times), 4-hydroxybenzoic acid (Ch2 > Ch1, 1.04 times) and 3-*O*-caffeoylquinic acid (Ch1 > Ch2, 1.33 times) (data not published). Cherries were characterized during the same period in which they were administered to the animals (Section 2.2).

### 2.2. Experimental Design

Animal experiments were conducted in accordance with the European Communities Council Directive (86/609/EEC) and approved by the Animal Ethics Review Committee for Animal Experimentation of the Universitat Rovira i Virgili and Generalitat de Catalunya (permission number 9495, FUE-2017-00499873). A total of 72 male Fischer 344/IcoCrl rats (7–8 weeks old, body weight 216–244 g) were purchased from Charles River Laboratories (Barcelona, Spain) and housed in pairs at 22 °C and 50% humidity under three photoperiods (light intensity 700 lx): L6 (short photoperiod; 6 h light/day), L12 (12 h light/day) and L18 (long photoperiod; 18 h light/day). Throughout the experiment, the rats were fed a standard chow diet (AO4, Panlab, Barcelona, Spain) and tap water ad libitum. After 4 weeks of photoperiod acclimatization, F344 rats in each photoperiod group were randomly subdivided into three groups. One group of F344 rats received chow diet, which was supplemented with 100 mg Ch1 dry weight (dw)/kg body weight (bw) (Ch1 group), other group received 100 mg Ch2 dw/kg (Ch2 group) and the control group (C group) received 21.2 mg glucose /kg bw and 21.2 mg fructose /kg bw to match the sugar contents of the diets between the three groups (*n* = 8 per group). The group size [22,23,24] and cherry dose [24] were selected based on the results of previous studies. During the four weeks of photoperiod acclimatization, rats were trained to lick a syringe with water, which allowed us to avoid oral gavage, thus reducing stress and improving animal welfare during the seven weeks of fruit supplementation. Fruit supplements were dissolved in tap water and administered by voluntary licking with a syringe. The experimenter visually confirmed that the full dose was administered. Rats were weighed every week, and food intake was measured in the last week. The weight (in grams) of the pellets of the standard diet was recorded when the food was provided and subtracted after 24 h to calculate how many grams of rat pellets were consumed. The eating pattern index (EPI) was calculated according to the calculations used by Ruiz de Azua et al. 2023 [16] and considers the kcal consumed as well as the fact that animals eat mainly during the hours of darkness: EPI (kcal/h): (kcal/day)/hours of darkness/day. Health checks were performed daily, and no welfare-related issues were observed.

**Figure 1:**
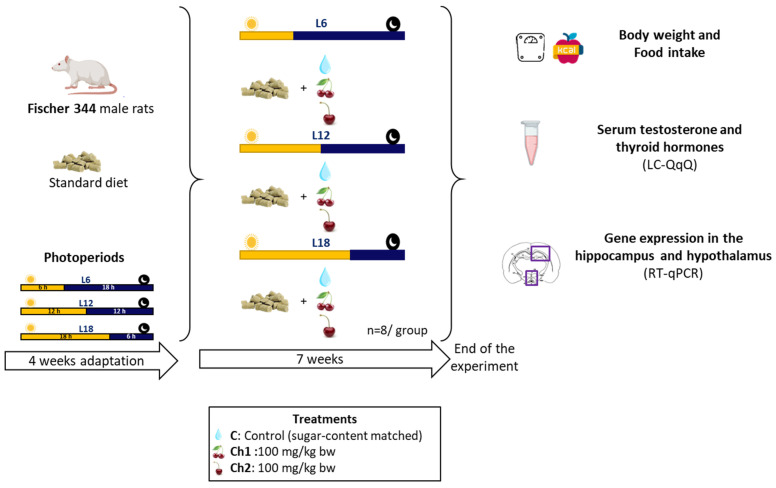
Experimental design and timeline. F344 rats were acclimatized to L6 (6 h light/day), L12 (12 h light/day) or L18 (18 h light/day) on a chow diet for four weeks. Rats in each photoperiod group (*n* = 24 per photoperiod) were randomly assigned to three groups. One group in each photoperiod was provided with chow and either Cherry 1 from Spain, Cherry 2 from Chile or a sugar-content-matched control. The experiment was completed after seven weeks of photoperiod and diet intervention.

At the end of the experiment, rats were deprived of food after supplementation with Ch1, Ch2 or C and were killed by decapitation 1 h later between ZT1 and ZT2 (i.e., 1 and 2 h after the beginning of the light phase, respectively). Paired testes weights were recorded, and brains were dissected and immediately frozen in liquid nitrogen and stored at −80 °C until use. The hypothalamus and hippocampus were dissected at −20 °C on the day of RNA extraction to maintain RNA integrity. Figure 1 shows a graphical representation of the experimental design used in this study.

### 2.3. High-Performance Liquid Chromatography Coupled to Triple Quadrupole (LC-QqQ)

Serum testosterone T3 and T4 concentrations were measured using high-performance liquid chromatography coupled with triple quadrupole (LC-QqQ) following the method and using the equipment described by Domenech-Coca et al. 2019 [25]. Samples were prepared by mixing 50 μL serum with 250 μL methanol containing the internal standard (2 ng/mL). The mixture was then vortexed and centrifuged for 5 min at 4 °C at 20,000× *g*. The supernatant was mixed with 700 μL of 0.1% formic acid in water and subjected to solid-phase extraction using an Oasis HLB 96-well plate (Waters, Milford, MA, USA) previously conditioned with methanol and 0.1% formic acid in Milli-Q water. The cartridge was washed with 0.1% formic acid in Milli-Q water and dried under a high vacuum. The compounds were eluted with 500 μL methanol. Samples were evaporated in a SpeedVac at 45 °C, reconstituted with 50 μL Milli-Q water: methanol (60:40, *v*/*v*) and transferred to a glass vial for analysis. Then, 5 µL of solution was injected into a spectrometry UHPLC 1290 Infinity II Series (Agilent Technologies, Santa Clara, CA, USA) coupled to a QqQ/MS 6490 Series (Agilent Technologies) using a C18 analytical column (Zorbax Eclipse, 150 × 2.1 mm, Agilent Technologies). The chromatographic separation was performed with a gradient (40% of B for 0.5 min, 40–80% of B for 10.5 min, 80–100% for 0.1 min, 100% for 1.1 min, 100–40% for 0.1, and 40% for 3.5 min); mobile phase A was water with 0.5% acetic acid and 10 mM ammonium acetate, while B was 100% methanol. The column temperature was set at 45 °C and the flow rate was 3 mL/min. Standards and internal standards (3,3′,5’-triiodo-L-thyronine-13C6 solution, L-thyroxine-d4 and testosterone d3 for T3 and T4 and testosterone, respectively) were dissolved in methanol. Calibration curves were constructed in the ranges 0.05–10 ng/mL for testosterone, 0.05–5 ng/mL for T3 and 1–100 ng/mL for T4.

### 2.4. Gene Expression Analysis in the Hypothalamus and Hippocampus

Approximately 70–80 mg of hypothalamus and hippocampus tissues were dissected from each rat brain, transferred to cell disruption buffer and homogenized with 1.5 mm homogenization beads (Triple-Pure™, Molecular Biology Grade Zirconium Beads) using the BeadBug microtube homogenizer. After homogenization, RNA extraction was performed according to the manufacturer’s protocol using the PARIS™ kit (Invitrogen, Thermo Fisher Scientific, Inc., Waltham, MA, USA). The RNA was quantified using a Nanodrop™ spectrophotometer (Nanodrop Lite; Thermo Fisher Scientific). RNA (500 ng) was used to synthesize cDNA using a High-Capacity cDNA Reverse Transcription Kit, and qPCR was performed using the Bio-Rad real-time system CFX96 to amplify gene expression levels with the following parameters: 40 cycles of 2 min at 50 °C, 5 min at 95 °C, 10 s at 95 °C and 30 s at 60 °C. mRNA levels were normalized to D-Box, and fold changes were calculated using the 2^−ΔΔCt^ method. The primers used to amplify these genes are listed in Supplementary Table S1.

### 2.5. Statistical Analysis

Data are expressed as mean ± standard deviation (SD). One-way ANOVA (Tukey’s post-hoc test) was used to determine the differences between the groups (*p* < 0.05). Two-way repeated-measures ANOVA (Tukey’s post-hoc test) analysis of Photoperiod x Fruit was used to assess the differences between groups (F being fruit and P being photoperiod effect, with PxF being the effect of the interaction). The interaction was considered statistically significant with a *p* value of *p* < 0.05. Statistical tests were performed using the SPSS Statistics 22 software (SPSS Inc., Chicago, IL, USA). Outliers were identified using SPSS using Tukey’s method and were removed before statistical analysis.

## 3. Results

### 3.1. Effects of Photoperiod on Body Weight, Eating Pattern Index and Serum Testosterone Levels in F344 Rats

The photoperiod responsiveness of F344 rats was assessed using body weight, EPI, testes weight, serum testosterone and the T4/T3 ratio (Table 1). After 11 weeks of photoperiod exposure, two-way ANOVA revealed a significant difference in body weight between the groups (P, *p* < 0.05), with a 6% lower body weight in L6-C than in L12-C (Tukey’s post hoc test, *p* = 0.06). Within the photoperiod groups, no significant difference was found in cherry consumption; however, L6-Ch1 showed a significant decrease in body weight compared with L12-Ch1 (*p* < 0.05). To assess eating behavior, EPI was calculated for all groups and represented as kilocalories consumed by the rats and the hours of darkness when the animals were active and fed (Table 1). EPI was significantly affected by the photoperiod as follows: L6-C < L12-C < L18-C (P, two-way ANOVA, *p* < 0.05). Similar to body weight, no differences were found in EPI between Ch1 or Ch2. 

No significant difference was found in paired testes weights (Table 1); however, two-way ANOVA revealed a significant interaction between photoperiod and cherry consumption for serum testosterone levels (P, PxF, two-way ANOVA, *p* < 0.05). Specifically, testosterone concentrations were significantly lower in L18-C than in L6-C and L12-C (Table 1). Ch1 increased serum testosterone levels in the L6-Ch1 and L12-Ch1 groups compared with their control groups (the L6-C and L12-C groups, respectively); however, no effects of cherry consumption were found in the L18 photoperiod. 

Circulating levels of thyroid hormones in serum (T4/T3 ratio) were affected by photoperiod and fruit (PxF, two-way ANOVA, *p* < 0.05), with the lowest T4/T3 ratio in L6-C compared with the other photoperiod control groups (Table 1). Moreover, Ch1 significantly increased the T4:T3 ratio in F344 rats exposed to the L6 photoperiod compared with L6-C animals (Table 1).

### 3.2. Hypothalamic Gene Expression Responds to Photoperiod

**Figure 2:**
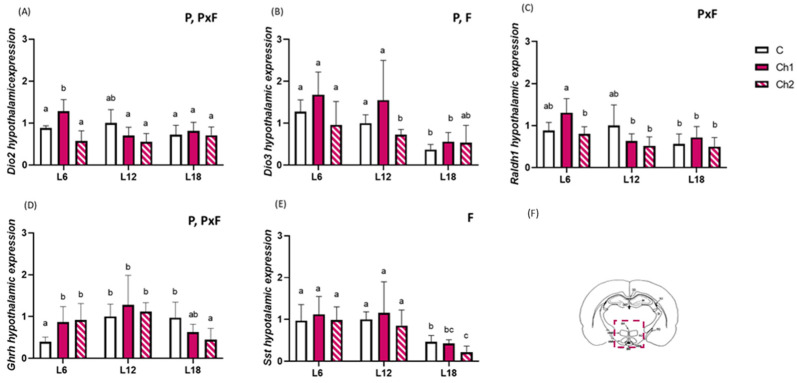
Hypothalamic (**A**) deiodinase 2 (*Dio2),* (**B**) deiodinase 3 (*Dio3*)*,* (**C**) retinaldehyde dehydrogenase 1 (*Raldh1)*, (**D**) growth hormone-releasing hormone *(Ghrh)* and (**E**) somatostatin *(Sst)* mRNA expression in Fisher 344 rats exposed to L6 (6 h light/18 h dark), L12 (12 h light/12 h dark) or L18 (18 h light/6 h dark) photoperiods for eleven weeks and administered vehicle (**C**), cherry 1 (Ch1, 100 mg/kg body weight) or cherry 2 (Ch2, 100 mg/kg body weight) during the last seven weeks. Values are expressed as mean ± standard deviation and normalized relative to the L12-C group (*n* = 8 per group). P, F and PxF indicate significant differences produced by photoperiod, fruit and the interaction between photoperiod and fruit, respectively (two-way ANOVA, *p* < 0.05). Different letters above the bars indicate significant differences by post-hoc Tukey’s test (one-way ANOVA, *p* < 0.05). (**F**) Schematic representation of the brain. The region included in the red square shows the analyzed brain region.

Previous studies have shown a range of photoperiodically regulated genes in the hypothalamus in F344 rats, including genes involved in thyroid hormone and retinoic acid signaling [26,27,28]. Here, we tested the key genes involved in photoperiodic time measurements and assessed whether they were also regulated by cherry consumption. Hypothalamic *Dio2* expression did not show photoperiod responsiveness in the control groups after 11 weeks of photoperiod exposure; however, Ch1 consumption significantly upregulated *Dio2* expression in F344 rats exposed to the L6 photoperiod compared with all other groups, except L12-C (Figure 2A; two-way ANOVA P, PxF *p* < 0.05). *Dio3* was downregulated in L18-C compared with the L6-C and L12-C groups and in L12-Ch2 compared with L12-C (Figure 2B) (P, F two-way ANOVA, *p* < 0.05). 

An interaction between photoperiod and fruit was found in the expression levels of *Retinaldehyde dehydrogenase 1* (*Raldh1*) (PxF, two-way ANOVA, *p* < 0.05); however, we did not find differences in photoperiod (Figure 2C). After Ch1 consumption, hypothalamic *Raldh1* expression levels were downregulated in the L12-Ch1 and L18-Ch1 groups compared with the L6-Ch1 group. 

Furthermore, mRNA expression levels of hypothalamic *Growth-hormone-releasing hormone* (*Ghrh*) and *Somatostatin* (*Sst*) were analyzed. *Ghrh* mRNA levels were strongly affected by photoperiod, with lower levels in L6-C than in L12-C and L18-C; however, this effect disappeared after the consumption of both cherries (L6-Ch1 and L6-Ch2 groups) (Figure 2D). Interestingly, *Ghrh* mRNA expression was downregulated by the consumption of Ch2 in L18, reaching expression levels similar to those in the L6-C group (Figure 2D). Two-way ANOVA revealed an effect of photoperiod and an interaction between photoperiod and fruit (two-way ANOVA; PxF, P, *p* < 0.05). *Sst* levels were downregulated in L18-C compared with in those L12-C and L6-C groups. Moreover, the Ch2 group showed downregulation of *Sst* mRNA compared with all other groups (two-way ANOVA, F, *p* < 0.05). Hence, we found that both genes not only respond to photoperiod but also to sweet cherry consumption within a specific photoperiod, as Ch2 exacerbated the downregulation of *Ghrh* and *Sst* in the L18 group.

### 3.3. Sweet Cherry Consumption Exerts a Hippocampal Neuroprotective Effect, Which Was Modulated by Specific Phenolic Hallmarks and Photoperiod

**Figure 3:**
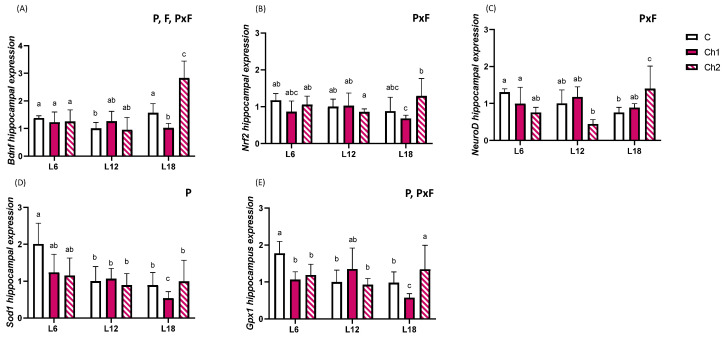
Hippocampal (**A**) brain-derived neurotrophic enzyme *(Bdnf)*, (**B**) nuclear factor erythroid-derived 2-like 2 (*Nrf2)*, (**C**) neuronal differentiation 1 (*NeuroD)*, (**D**) superoxide dismutase type 1 (*Sod1)* and (**E**) glutathione peroxidase 1 (*Gpx1)* mRNA expression in Fisher 344 rats exposed to L6 (6 h light/18 h dark), L12 (12 h light/12 h dark), or L18 (18 h light/6 h dark) photoperiods for eleven weeks and administered vehicle (**C**), cherry 1 (Ch1, 100 mg/kg body weight) or cherry 2 (Ch2, 100 mg/kg body weight) during the last seven weeks. Values are expressed as mean ± standard deviation and normalized relative to the L12-C group (*n* = 8 per group). P, F and PxF indicate significant differences produced by photoperiod, fruit and the interaction between photoperiod and fruit, respectively (two-way ANOVA, *p* < 0.05). Different letters above the bars indicate significant differences by post-hoc Tukey’s test, (one-way ANOVA, *p* < 0.05).

Next, we investigated the possible neuroprotective effects of seasonal consumption of sweet cherries with different phenolic hallmarks on gene expression markers in the hippocampus. Growing evidence suggests that the consumption of fruit is associated with neuroprotective effects [29,30], but reports on the effect of photoperiod on neuroprotection are less common. *Bdnf* mRNA levels were modulated by P, F and PxF (two-way ANOVA, *p* < 0.05), with Ch2 consumption strongly upregulating *Bdnf* expression compared with all other groups (Figure 3A). *Nrf2* expression levels were affected by fruit consumption and photoperiod (PxF, *p* < 0.05). The L18-Ch2 group showed higher expression levels compared with the L18-Ch1 group (Figure 3B). *Neuronal differentiation 1* (*NeuroD*) was regulated by photoperiod, with lower *NeuroD* expression in the L18-C group than in the L6-C group; however, Ch2 consumption significantly upregulated *NeuroD* expression compared with all other groups (Figure 3C). In addition, *superoxide dismutase type 1* (*Sod1*) was significantly affected by photoperiod and was upregulated in the L6-C group compared with the L12-C and L18-C groups. Consumption of Ch1 significantly downregulated *Sod1* levels, whereas Ch2 consumption maintained the expression levels of *Sod1* (Figure 3D). *Glutathione peroxidase 1* (*Gpx1*) showed a similar expression pattern to *Sod1*; however, Ch2 consumption significantly upregulated *Sod1* expression compared with L18-C and Ch1 (Figure 3E).

## 4. Discussion

In this study, we investigated the effects of the consumption of seasonal cherry, which is rich in anthocyanins, on the activation of neuroprotective pathways in the hippocampus of photoperiod-sensitive F344 rats. Two cherries obtained from different geographical regions were selected for the study in order to obtain cherries with different chemical compositions. We showed that the markers of neuroprotection are strongly regulated by photoperiod and/or seasonal fruit consumption. 

The consumption of fruit is associated with several beneficial health effects, including the reduction of blood pressure and cell damage through its antioxidant effects [29,30]. Phenolic compounds are one of the main compounds responsible for the health benefits of fruit, which can have antioxidant and anti-inflammatory effects [31]. Specifically, it has been reported that anthocyanins, found in cherry and blueberry for example, have excellent antioxidant properties, improving oxidative stress and neurodegeneration, among others [32]. Importantly, phenolic compounds can cross the blood–brain barrier [33,34]. The phenolic compounds quercetin and naringin have been shown to promote neuroprotection in the dentate gyrus of the hippocampus [35], which is the main brain region that hosts neurogenic niches [36]. In support, the consumption of blueberries, a fruit rich in phenolic compounds, exerts positive effects on the brain by enhancing memory and learning in animals and humans [37]. 

Firstly, to evaluate the photoperiod responsiveness of the F344 rats in this study, we analyzed physiological parameters (body weight, eating pattern), photoperiod-responsive hormones and photoperiod-regulated genes in the hypothalamus. As expected, we found that body weight and feeding behavior responded to the photoperiod. These results are consistent with previous studies in F344 rats showing that body weight and food intake are strongly regulated by photoperiod, with lower body weight and food intake in short photoperiods (L6) compared with long photoperiods (L18) [2,16,22]. However, compared with these studies, the effect of photoperiod on body weight in our study was more subtle. Differences between studies could be attributed to photoperiod refractoriness, as this study was carried out during eleven weeks of photoperiods. Refractoriness is a physiological response of seasonal species such as hamsters and sheep to a change in photoperiod that spontaneously reverts to the initial state, even if the photoperiod remains unchanged [38]. Another reason could be that different sub-strains of F344 respond differently to photoperiod; for example, F344/NHsd rats are more sensitive to photoperiod than F344⁄NCrHsd [39]. Interestingly, Ch1 exacerbated the photoperiod effect by decreasing body weight under a short photoperiod. 

A shared mechanism of photoperiod responsiveness among different seasonal species is the change in hypothalamic thyroid hormone availability, depending on the photoperiod of exposure [1]. Here, we show that hypothalamic thyroid signaling was not only affected by photoperiod but also varied depending on cherry consumption. It has been widely described that the gene expression of *Dio2* and *Dio3* enzymes in tanycytes regulates hypothalamic thyroid hormone availability by converting between biologically active T3 and inactive T4 [40]. In our study, only hypothalamic *Dio3* expression was regulated by photoperiod, with a downregulation in the long photoperiod as expected [28,41,42]. Species-specific differences in *Dio2* and *Dio3* expression have been discussed previously [40], although previous studies in F344 rats indicated a photoperiod response with upregulation of *Dio2* in long photoperiods [2,27]. However, in these studies, the maximum photoperiod exposure was four weeks, whereas, in our study, rats were exposed to a photoperiod for eleven weeks, and photoperiod refractoriness could not be ruled out. 

Intra-hypothalamic thyroid hormone metabolism plays an important role in seasonal response [43], but the seasonal pattern of serum thyroid hormone levels remains controversial. According to a clinical study in 700,000 people, only thyroid-stimulating hormone levels showed a seasonal rhythm, whereas T3 and T4 levels were not affected [44]. Nevertheless, another study with over 7000 volunteers showed monthly changes in serum T3 and T4 levels [45]. It has been suggested that diets rich in fruits (i.e., rich in (poly)phenols) have beneficial effects on thyroid hormones in population studies [46]. Here, we show that the T4/T3 ratio increased with the consumption of Ch1 under short photoperiods, suggesting that the effect of cherry consumption on the thyroid hormones depends on photoperiod in F344 rats and on chemical composition. Different studies have shown that phenolic compounds can act as activators or inhibitors of the thyroperoxidase enzyme, which is involved in thyroid hormone synthesis [47,48]. Thus, the effect of Ch1 could be the result of the sum of all the inhibitory and activating actions produced by several phenolic compounds. 

RALDH1 is involved in retinoic acid synthesis, and its absence is associated with decreased food intake and body weight in F344 rats [26]. Our results contrast with those of other studies carried out in F344 rats exposed to photoperiods for three and fourteen days, where *Raldh1* showed a rapid and strong photoperiod effect and was downregulated under short photoperiods [26]. The differences between the two studies could be attributed to the duration of photoperiod exposure, as photoperiod refractoriness could be involved in this pathway. The growth hormone axis is seasonally regulated, and its increase is associated with an increase in day length in seasonal species [49]. Growth hormone is involved in the promotion of anabolic functions when nutrients are available. In contrast, SST inhibits its activity as a feedback regulatory mechanism [50]. Different nutrients, such as amino acids, vitamins and minerals, play a role in the regulation of the growth axis [50], and it has been suggested that a Mediterranean diet, rich in fruits and vegetables, is correlated with a peak in growth hormone in humans [51]. In this study, we demonstrated that *Ghrh* and *Sst* expressions were regulated by photoperiod in F344 rats and that the consumption of Ch2 was associated with decreased expression. These effects could be attributed to the fact that Ch2 contained more flavonols and hydrobenzoic acid derivatives (unpublished data). 

In mammals, reproduction is strongly modulated by photoperiod [52]. For example, the testes weight of F344 rats decreases during the first weeks of a short photoperiod, although these changes disappear after 10 weeks of photoperiod exposure [53]. These findings agree with the results observed in the present study, since no differences were observed in testes mass after 11 weeks of photoperiod exposure. We observed that testosterone levels are modulated by Ch1 but not Ch2 in the L6 and the L12 photoperiods, which could be linked to the different compositions of the two sweet cherries. These differential effects may be due to Ch1′s higher concentration of cyanidin derivatives and catechin than Ch2 (unpublished data), which may be the result of the harvesting conditions in different geographical areas. Moreover, sex hormones have been correlated with neuroprotection in neuronal cell models; however, in the presence of oxidative stress, they increase cell loss in both neuronal and glial cells [54]. This evidence allowed us to evaluate photoperiod responsiveness in this study to later focus on the neuroprotective role of cherries with different origins and compositions. 

Among the factors that regulate hippocampal homeostasis, diets rich in fruits and vegetables have been associated with improvements in cognition and working memory [55]. For instance, the consumption of tart cherry in aged F344 rats decreases the expression of neuroinflammatory markers such as NADPH oxidase and cyclooxygenase 2 [56]. Other studies have observed that phenolic compounds, such as quercetin and kaempferol, activate key proteins involved in neuroprotection in the hippocampus, including NRF2 and BDNF. NRF2 acts in the nucleus by inducing the transcription of genes containing a specific region called the antioxidant response element (ARE) [57]. This signaling promotes the expression of antioxidant enzymes such as GPX1 and SOD by reducing cell damage and the production of reactive oxygen species [35]. Neuroprotection in the brain is correlated with the antioxidant enzymatic activity of a subset of proteins, which can be stimulated by external factors such as the intake of fruits rich in bioactive compounds [58]. Additionally, thyroid hormones have been observed to play an important role in adult neurogenesis in the hippocampus, promoting cell survival and upregulating the expression of genes involved in cell differentiation, such as NeuroD [59]. Considering that thyroid hormones play a pivotal role in generating seasonal rhythms, it is feasible to hypothesize that neuronal function in the hippocampus is affected by photoperiod. In support, we recently showed that a short photoperiod impaired memory in F344 rats [22]. In the current study, hippocampal *Bdnf*, *Nrf2*, *NeuroD* and *Gpx1* expression levels were upregulated when rats consumed Ch2 in the L18 photoperiod, promoting hippocampal neuroprotection and stimulating the expression of genes involved in antioxidant signaling, suggesting that cherry effects are regulated by photoperiod. Given that Ch1 did not exert any effects, the compositions of the sweet cherries must be pivotal in their different effects. The phenolic compositions of the two cherries were different: Ch1 had a higher content of total polyphenol, anthocyanin and flavanol than Ch2, while Ch2 showed a higher flavonol content than Ch1 [11]. In addition, Ch2 had a higher content of quercetin, quercetin derivatives and kaempferol derivatives than Ch1 (data not published). As mentioned above, quercetin and kaempferol can activate proteins involved in hippocampal neuroprotection [57]. Thus, these flavonols may be involved in the neuroprotective effects of Ch2. Previously, we showed that lipid peroxidation markers due to free radicals are reduced by the consumption of both cherries in L18, with one of the cherries being more effective in lowering these markers, highlighting that the composition of the cherry influences the differential response [11]. Moreover, the effects could be different from one organ to another because, in the case of the brain, the blood–brain barrier is highly selective. It has been observed that metabolites derived from the digestion of (poly)phenols cross the blood–brain barrier depending on their molecular structure, and their metabolism in the endothelial layer produces a variety of metabolites with additional bioactive properties [33]. While a previous study did not show a photoperiod response in *Bdnf* mRNA expression in the hippocampus of hamsters exposed to short or long photoperiods [60], clinical studies have shown that circulating markers of neuroprotection, such as BDNF, show a seasonal pattern [61]. Moreover, we showed that testosterone levels were strongly increased by cherry consumption in short and intermediate photoperiods, and it has been previously suggested that the consumption of anthocyanins is associated with a reduction in oxidative-stress-induced testosterone deficiency [62]. To the best of our knowledge, this is the first study showing photoperiod-dependent regulation of neuroprotective markers in the hippocampus with a specific type of sweet cherry.

## 5. Conclusions

In conclusion, seasonal consumption of sweet cherry with a specific composition could play an important role in the hippocampal activation of neuroprotection in a photoperiod-dependent manner by increasing the expression markers of neuroprotection in a long photoperiod. However, further research is needed to elucidate how the time of year could affect the benefits of fruit consumption in humans by improving neuroprotective markers, including clinical trials and bioavailability studies to elucidate the possible phenolic metabolites responsible for the observed effects.

## Figures and Tables

**Table 1:** Biometric parameters and blood hormone levels of Fisher 344 rats exposed to different photoperiods and administered vehicle or cherries for seven weeks.

Parameters	L6 Photoperiod			L12 Photoperiod			L18 Photoperiod			2wA
	C	Ch1	Ch2	C	Ch1	Ch2	C	Ch1	Ch2	
Final BW (g)	367.7 ± 13.7 ^ab^	369.5 ± 20.0 ^b^	370.8 ± 21.3 ^ab^	389.8 ± 25.4 ^a^	393.7 ± 14.9 ^a^	381.8 ± 17.1 ^ab^	383.7 ± 27.8 ^a^	387.8 ± 20.7 ^ab^	376.1 ± 24.0 ^ab^	P
EPI (kcal/h of dark)	3.00 ± 0.04 ^a^	3.16 ± 0.19 ^a^	3.06 ± 0.25 ^a^	4.62 ± 0.27 ^b^	4.68 ± 0.27 ^b^	4.48 ± 0.26 ^b^	9.15 ± 0.22 ^c^	9.02 ± 0.22 ^c^	9.19 ± 0.47 ^c^	P
Testosterone (ng/mL)	2.98 ± 0.72 ^a^	4.16 ± 1.02 ^b^	3.45 ± 1.43 ^ab^	2.95 ± 0.98 ^a^	4.20 ± 1.09 ^b^	3.68 ± 1.83 ^ab^	1.63 ± 1.0 ^c^	1.86 ± 0.98 ^c^	2.43 ± 1.39 ^ac^	P, PxF
Testes (g)	3.08 ± 0.16	3.13 ± 0.14	3.15 ± 0.12	3.15 ± 0.13	3.20 ± 0.1	3.26 ± 0.14	3.05 ± 0.17	3.2 ± 0.15	3.2 ± 0.17	
T4/T3	42.46 ± 2.64 ^a^	50.68 ± 4.89 ^b^	47.76 ± 4.85 ^ab^	48.31 ± 4.41 ^b^	47.45 ± 5.90 ^ab^	47.89 ± 5.40 ^b^	51.25 ± 6.14 ^b^	51.97 ± 6.47 ^b^	47.6 ± 5.22 ^ab^	PxF

Fisher 344 rats were exposed to L6 (6 h light/day), L12 (12 h light/day) or L18 (18 h light/day) photoperiods for eleven weeks and administered vehicle (C), cherry 1 (Ch1, 100 mg/kg body weight) or cherry 2 (Ch2, 100 mg/kg body weight) during the last 7 weeks of the experiment. Values are expressed as mean ± standard deviation (*n* = 8/group). P and PxF indicate significant differences in photoperiod and interactions between photoperiod and fruit, respectively (two-way ANOVA, 2wA, *p* < 0.05). Different letters indicate significant differences among groups for each parameter (one-way ANOVA, post-hoc Tukey’s test, *p* < 0.05; one-way ANOVA, post-hoc Tukey’s test, *p* < 0.05-*p* < 0.01). Abbreviations: body weight (BW), eating pattern index (EPI), triiodothyronine (T3), thyroxine (T4).

## Data Availability

Data are contained within the article and the Supplementary Materials.

## Supplementary Materials

The following supporting information can be downloaded at https://www.mdpi.com/article/10.3390/antiox13010072/s1. Table S1: Primer nucleotide sequences used for real-time quantitative PCR.
