# Supplementary material for: Cherries with Different Geographical Origins Regulate Neuroprotection in a Photoperiod-Dependent Manner in F344 Rats
Source: Antioxidants (Basel). 2024 Jan 3;13(1):72. doi: 10.3390/antiox13010072 (PMC10812723; doi:10.3390/antiox13010072)
Supplement: Supplementary file 1 [file antioxidants-13-00072-s001.zip › antioxidants-2773569-supplementary.pdf]

Supplementary material

Table S1. Primer nucleotide sequences used for real-time quantitative PCR.

Genes	Forward (5' to 3')	Reverse (5' to 3')
Bdnf	GTCTGTCTGTAAGGGCTAGAATG	GTCTCCTATGAAGCCACCTAATC
Dio2	GCGACCTGACCACCTTTTACTAG	GCAGCACATCGGTCCTCTTG
Dio3	AATTGCAGAGGGGCTCGAAA	TTCCTTTGGTCCTGAAGCCC
Ghrh	TTAGGGTCTGGACATCACTGG	GCCCACACTCTGTCCAAATG
Gpx1	CAGTCCACCGTGTATGCCTT	GTAAAGAGCGGGTGAGCCTT
NeuroD	AGCCCCCTAACTGATTGCAC	CCCGGGAATGGTGAAACTGA
Nrf2	CTCTCTGGAGACGGCCATGACT	CTGGGCTGGGGACAGTGGTAGT
Raldh1	ACGTGGAAGAAGGGGACAAGGCTG	GCAAAGACTTTCCCACCATTGAGTGCC
Sod1	TAACTGAAGGCGAGCATGGG	TCCCAATCACACCACAAGCC
Sst	CCCCAGACTCCGTCAGTTTC	AACGCAGGGTCTAGTTGAGC

Abbreviations: *Bdnf* (brain derived neurotrophic enzyme); *Dio2* (deiodinase 2); *Dio3* (deiodinase 3); *Ghrh* (growth hormone realising hormone); *Gpx1* (glutathione peroxidase 1); *NeuroD* (neuronal differentiation 1); *Nrf2* (nuclear factor erythroid-derived 2-like 2); *Raldh1* (retinaldehyde dehydrogenase 1); *Sod1* (superoxide dismutase type 1); *Sst* (somatostatin)
